# Chemoprotective Effects of Xanthohumol against the Carcinogenic Mycotoxin Aflatoxin B1

**DOI:** 10.3390/foods10061331

**Published:** 2021-06-09

**Authors:** Alja Štern, Veronika Furlan, Matjaž Novak, Martina Štampar, Zala Kolenc, Katarina Kores, Metka Filipič, Urban Bren, Bojana Žegura

**Affiliations:** 1 National Institute of Biology, Department of Genetic Toxicology and Cancer Biology, Večna Pot 111, SI-1000 Ljubljana, Slovenia; alja.stern@nib.si (A.Š.); matjaz.novak@nib.si (M.N.); martina.stampar@nib.si (M.Š.); metka.filipic@nib.si (M.F.); 2 Faculty of Chemistry and Chemical Technology, University of Maribor, Smetanova 17, SI-2000 Maribor, Slovenia; veronika.furlan@um.si (V.F.); zala.kolenc@um.si (Z.K.); katarina.kores@um.si (K.K.); 3 Faculty of Mathematics, Natural Sciences and Information Technologies, University of Primorska, Glagoljaška 8, SI-6000 Koper, Slovenia

**Keywords:** xanthohumol, isoxanthohumol, 8-prenylnaringenin: 6-prenylnaringenin, aflatoxin B1, aflatoxin B1 exo-8,9-epoxide, genotoxicity, cytotoxicity, chemoprotective, antigenotoxic

## Abstract

The present study addresses the chemoprotective effects of xanthohumol (XN), a prenylated flavonoid found in the female inflorescences (hops) of the plant *Humulus lupulus* L., against the carcinogenic food contaminant aflatoxin B1 (AFB1). The chemical reactions of XN and its derivatives (isoxanthohumol (IXN), 8-prenylnaringenin (8-PN), and 6-prenylnaringenin (6-PN)) with the AFB1 metabolite, aflatoxin B1 exo-8,9-epoxide (AFBO), were investigated *in silico*, by calculating activation free energies (ΔG^‡^) at the Hartree–Fock level of theory in combination with the 6-311++G(d,p) basis set and two implicit solvation models. The chemoprotective effects of XN were investigated *in vitro* in the metabolically competent HepG2 cell line, analyzing its influence on AFB1-induced cytotoxicity using the MTS assay, genotoxicity using the comet and γH2AX assays, and cell cycle modulation using flow cytometry. Our results show that the ΔG^‡^ required for the reactions of XN and its derivatives with AFBO are comparable to the ΔG^‡^ required for the reaction of AFBO with guanine, indicating that XN, IXN, 8-PN, and 6-PN could act as scavengers of AFBO, preventing DNA adduct formation and DNA damage induction. This was also reflected in the results from the *in vitro* experiments, where a reduction in AFB1-induced cytotoxicity and DNA single-strand and double-strand breaks was observed in cells exposed to combinations of AFB1 and XN, highlighting the chemoprotective effects of this phytochemical.

## 1. Introduction

Nutrition and diet are fundamentally involved in human cancer etiology and prevention. The human diet is highly complex and versatile, containing numerous potentially mutagenic and carcinogenic ingredients, compounds, and contaminants [1], as well as various antioxidants and potentially chemoprotective and anticarcinogenic bioactive chemicals [2,3,4,5]. Prenylated flavonoids comprise a group of dietary phytochemicals with various beneficial health-related effects [6]. One of the best characterized prenylated flavonoids is xanthohumol (XN; 3′-[3,3-dimethylallyl]-2,4,4′-trihydroxy-6′-methoxychalcone), found in the female inflorescences (hops) of the plant *Humulus lupulus* L. (*Cannabinaceae*) [7]. The main dietary source of XN is beer, as hops are primarily used for flavoring, bittering, and preservation of beer, which dependent on beer type, contains XN in the range of 0.03–0.39 mg/L and total prenylated flavonoids, mainly the flavone isoxanthohumol (IXN), at concentrations of up to 9.47 mg/L [8]. XN spontaneously isomerizes into IXN and is transformed into several bioactive metabolites (manly O-glucuronides of XN and IXN, 8-prenylnaringenin (8-PN), and 6-prenylnaringenin (6-PN)) by hepatic metabolism and gut microbiota [7,9]. 6-PN and 8-PN are also present in beer in low amounts, at concentrations up to 0.17 mg/L and 0.06 mg/L, respectively [8]. Some brands also offer XN enriched beers, which can be achieved by prevention of XN isomerization through specialized brewing techniques and the late addition of hops at high quantities, which can result in XN concentrations of more than 10 mg/L in the finished beer [10]. 

XN is a potent antioxidant [11] and has shown protective effects against oxidative DNA damage [7,12], which has even been confirmed in a placebo-controlled human intervention trial study [13]. However, it also exerts chemoprotective effects via other mechanisms. In addition to antioxidative properties, XN inhibits the activity of cytochrome P450 enzymes involved in pro-carcinogen activation and promotes detoxification processes. Moreover, it was reported to have anti-inflammatory (inhibition of nuclear factor kappa-light-chain-enhancer of activated B cells (NF-κB) activation), apoptosis promoting (reducing the mitochondrial inner membrane potential), and anti-angiogenic properties (inhibition of the pro-angiogenic NF-κB and Protein kinase B (Akt) pathways) [7,12]. It has also been shown to exert protective effects against chemically induced mutagenesis [14] and DNA damage [15,16,17], and is a promising anti-cancer agent, potentially preventing various cancers, including leukemia, pancreatic, lung, colon, thyroid, breast, prostate, cervical, and ovarian cancers, glioblastoma, and hepatocellular carcinoma (HCC) [6].

Although difficult to accurately determine, because of the complexity and versatility of factors involved, the proportion of cancers attributable to diet and diet-related factors is estimated to be about 20% [18,19]. To date, few convincing links have been established between diet-related factors and cancer risk [20]. One of the most established is the relationship between the widely distributed mycotoxins, aflatoxins, and an increased risk of liver cancer [21]. Aflatoxin B_1_ (AFB1) is the most potent among them and is one of the most carcinogenic naturally occurring substances known [22]. It has been extensively studied and associated with HCC in humans and animals. It was classified as a human carcinogen (Group 1) by the International Agency for Research on Cancer (IARC) in 1993 [23]. It has been shown to be mutagenic, clastogenic, and aneugenic, and to cause unscheduled DNA synthesis, sister chromatid exchange, and epigenetic alterations in many model systems [22,24].

AFB1 is produced by the fungi *Aspergillus flavus* and *Aspergillus parasiticus*, common contaminants in food and feed commodities like maize, peanuts, cottonseed, tree nuts, rice, various spices, and a variety of other foods [25]. Thus, dietary intake is the primary, non-occupational route of exposure for humans. The average daily intake of AFB1 in developed countries is in the range of nanograms per day [26], while in countries where maize and peanuts are dietary staples, the daily intake is in the range of micrograms per day [27]. Following consumption, AFB1 is metabolized by cytochrome P450 enzymes (CYP450) in the liver, to the ultimate carcinogen aflatoxin-8,9-epoxide (AFBO), which is highly electrophilic and can spontaneously react with amines in proteins and nucleic acids, causing the formation of endogenous and different AFB1-specific DNA adducts [26]. AFBO predominantly interacts with guanine to form the pro-mutagenic trans-8,9-dihydro-8-(N7-guanyl)-9-hydroxyaflatoxin adduct (AFB1-N7-Gua), which can be further converted to the AFB1-formamidopyrimidine adduct (AFB1-FAPy), potentially leading to G-T transversion mutations found with high frequency in the *P53* tumor suppressor gene in HCC from populations exposed to high levels of dietary AFB1 [28,29].

The present study investigates the chemoprotective effects of the potent antioxidant XN against AFB1 *in silico* and *in vitro*. To elucidate the chemoprotective mechanisms of XN *in silico*, activation free energies (ΔG^‡^) for the chemical reactions of AFBO with XN and its derivatives IXN, 8-PN and 6-PN, were calculated at the Hartree−Fock (HF) level of theory in combination with the 6-311++G(d,p) basis set and two implicit solvation models. The chemoprotective effects of XN against AFB1-induced cytotoxicity and genotoxicity were further explored *in vitro* in human hepatocellular carcinoma HepG2 cells. The MTS assay was applied to determine XN-mediated protection against AFB1-induced cytotoxicity, protection against AFB1-induced genotoxicity was determined using the comet assay and the γH2AX assay, and the influence of XN on AFB1-induced cell cycle modulation was investigated with Hoechst 33342 staining.

## 2. Materials and Methods

### 2.1. Chemicals

Xanthohumol (XN), Aflatoxin B1 (AFB1), dimethylsulphoxide (DMSO), Benzo(a)pyrene (BaP), Minimal Essential Medium Eagle (MEM), NaHCO_3_, non-essential amino acids (NEAA), sodium pyruvate ethylenediaminetetraacetic acid (EDTA), NaCl, NaOH, and phenazine methosulfate (PMS) were all purchased from Sigma-Aldrich (St. Louis, MO, USA). Penicillin/streptomycin, phosphate-buffered saline (PBS), ethanol, fetal bovine serum, and L-glutamine were from PAA Laboratories (Toronto, Canada). Triton X-100 was from Thermo Fisher Scientific (Pittsburgh, PA, USA). Hoechst 33258, trypsin, low melting point agarose (LMP), and normal melting point agarose (NMP) were from Invitrogen (Waltham, MA, USA). The CellTiter96^®^ AQueous cell proliferation assay (3-(4,5-dimethylthiazol-2-yl)-2,5-diphenyltetrazolium bromide; MTS) was from Promega (Madison, WI, USA). Etoposide (ET) was from Santa Cruz Biotechnology (Dallas, TX, USA). GelRed Nucleic Acid Stain was from Biotium, (Fremont, CA, USA). Tris was from Merck (Darmstadt, Germany). Anti-γH2AX pS139, FITC conjugate human recombinant antibodies were from Miltenyi Biotec GmbH (Bergisch Gladbach, Germany). All other reagents were of the purest grade and solutions were made using Milli-Q water. Stock solutions of XN (70 mM) and AFB1 (3.2 mM) for the *in vitro* studies were prepared in DMSO and stored at −20 °C.

### 2.2. Computational Analysis of Alkylation Reactions between XN, IXN, 8-PN, and 6-PN and the Carcinogenic Metabolite of AFB1-AFBO

The Hartree-Fock (HF) method in combination with the flexible basis sets 6-311++G(d,p) was employed to predict the activation barriers of reactions involving XN and its derivatives IXN, 8-PN, and 6-PN with the ultimate carcinogen AFBO, for which kinetic experiments have not been conducted yet. 

Because the studied alkylation reactions do not take place *in vacuo*, solvation effects were incorporated by the self-consistent reaction field (SCRF) method of Tomasi et al. [30] and the Langevin dipole (LD) model of Florian and Warshel [31]. Quantum-mechanical calculations of activation free energies (ΔG^‡^) were performed with the Gaussian 16 on the CROW cluster located at the National Institute of Chemistry in Ljubljana [32,33]. To evaluate computational results, the obtained ΔG^‡^ for the reactions of AFBO with XN, and its derivatives IXN, 8-PN, and 6-PN were compared to the experimental ΔG^‡^ barriers for the reaction of AFBO with guanine. Experimental ΔG^‡^ for the reaction of AFBO with guanine was obtained from the experimentally determined rate constant *k* based on the transition state theory of Eyring (1) where *h* represents the Planck constant, *k_B_* the Boltzmann constant, and *T* the absolute temperature [34].

(1) $$k=\frac{k_{B}T}{h}e^{\left(-\frac{\Delta G^{‡}}{k_{B}T}\right)}$$

**Figure A1:**
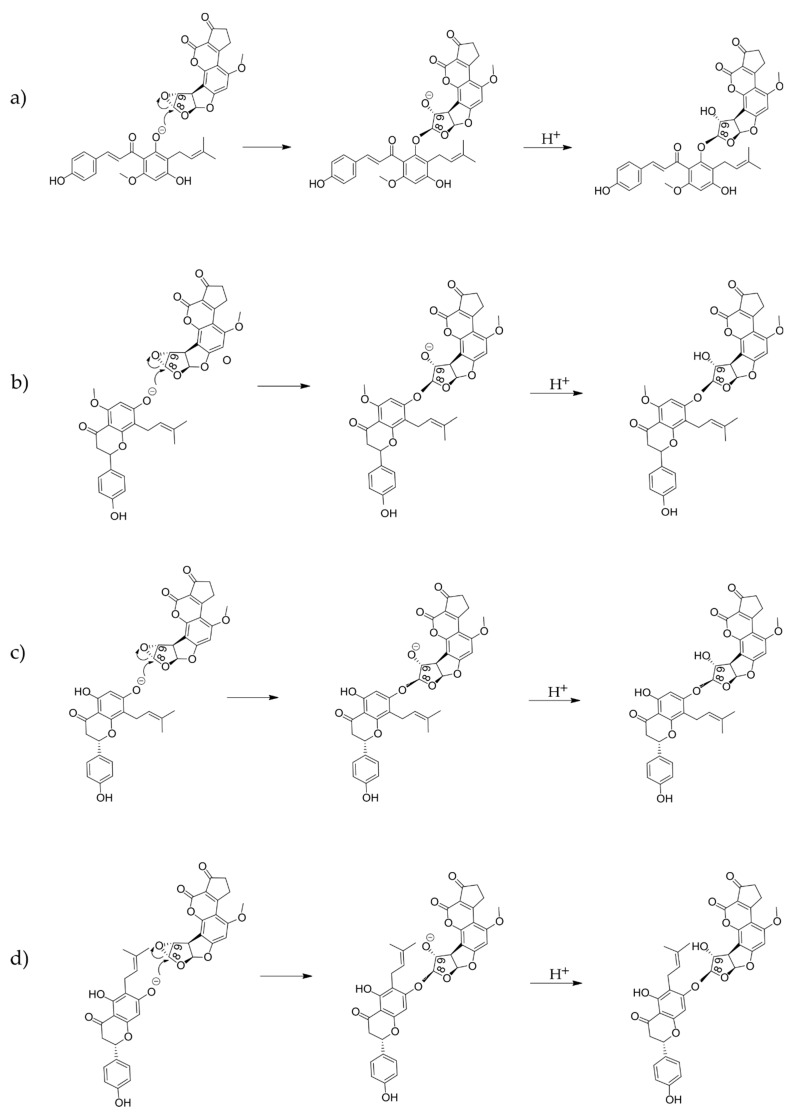
The reaction mechanisms proposed for the reactions of AFBO with (**a**) XN, (**b**) IXN, (**c**) 8-PN, and (**d**) 6-PN.

The transition state theory assumes that reactants and transition states form a thermal equilibrium. The ΔG^‡^ barriers for the investigated chemical reactions represent the free energy difference between the transition state and the reactant state structure. To obtain ΔG^‡^ of these reactions, the reactant and transition state structures had to be located. Geometry optimizations and vibrational analysis were carried out to obtain reactant structures determined by the local minimum on the potential energy surface. A relaxed potential surface scan was then performed [35] to discover approximate structures of the transition states. These approximate structures were chosen as the starting points for the Berny algorithm [36] and vibrational analysis that provided the optimized transition state structures [37]. XN, IXN, 8-PN, and 6-PN were studied in their nucleophilic (anionic) forms at a physiological pH of 7.4. The pKa values for the most nucleophilic oxygen atoms of XN and its derivatives were predicted with the MarvinSketch software package (18.5.0, ChemAxon, Budapest, Hungary ) [38]. The proposed molecular mechanisms presented in Figure A1 (Appendix A) were drawn using the ChemDraw program. Visual representations of reactant and transition state structures in Figure 3 were prepared in Avogadro [39].

### 2.3. Cell Culture for the In Vitro Assessment of XN Protective Effects against AFB1

HepG2 cells, from American Type Culture Collection ((ATCC), Manassas, VA, USA) were cultured in MEM medium, supplemented with FBS (10%), NEAA (1%), sodium pyruvate (1 mM), L-glutamine (2 mM), NaHCO_3_ (2.2 g/L), and penicillin/streptomycin (100 IU/mL), at 37 °C and 5% CO_2_. For the experiments, cells were seeded on assay-specific culture plates at specific densities and allowed to adhere overnight. The growth medium was replaced the next day with fresh growth medium containing XN (0.01, 0.1, 1, and 10 µM), AFB1 (10, 20, and 30 µM), and combinations of AFB1 (30 µM) and XN (0.01, 0.1, 1, and 10 µM). Cells were exposed to the tested compounds for 24 h. The solvent (DMSO) concentration in the medium was adjusted to 1% in test samples and solvent control groups. In all experiments, a solvent control (growth medium containing 1% DMSO), negative control (growth medium), and assay-specific positive controls were included. 

### 2.4. Changes in Cell Viability—The MTS Assay

The chemoprotective effects of XN against AFB1-induced cytotoxicity were evaluated using the MTS assay as described by Hercog et al. [40]. ET (25 μg/mL) was used as a positive control. The program GraphPad Prism 9 (GraphPad Software, San Diego, CA, USA) was used for statistical analysis and data visualization. ANOVA (one-way analysis of variance) and Dunnet’s multiple comparison test were used to determine statistically significant differences in cell viability between the samples.

### 2.5. Induction of DNA Strand Breaks—The Alkaline Comet Assay

The chemoprotective effects of XN against AFB1-mediated induction of DNA single-(SSBs), double-strand breaks (DSBs), and/or alkali labile sites (ALS), was analyzed with the alkaline comet assay according to the description by Žegura and Filipič [41], with minor modifications. Cells were seeded at a density of 80,000 cells/well on 12 well plates (Corning, Corning Costar Corporation, NY, USA). For the image analysis, cell nuclei were stained with GelRed according to the manufacturer’s protocol, and images were acquired and analyzed using a fluorescence microscope (Eclipse 800, Nikon, Tokyo, Japan) and the software Comet IV (Perceptive Instruments Ltd., Haverhill, UK). BaP (30 μg/mL) was used as a positive control. Three independent experiments were performed, wherein fifty nuclei were analyzed per experimental point. The Kruskal–Wallis nonparametric test and Dunn’s multiple comparison test (GraphPad Prism 9) were used to assess statistically significant differences in the percentage of tail DNA between the tested cell populations. 

### 2.6. Induction of Double Strand Breaks—The γH2AX Assay

The induction of DNA double-strand breaks (DSBs) was assessed by measuring phosphorylation of the histone H2AX using flow cytometry (MACSQuant Analyzer 10; Miltenyi Biotec, Bergisch Gladbach, Germany) as described by Hercog et al. [40] with minor modifications. The cells (750,000 cells/plate) were seeded on T25 plates (Corning, Corning Costar Corporation, NY, USA) and left to attach overnight. After 24 h of exposure to the tested compounds, the cells were collected and fixed with ethanol (70%) and stored at −20 °C until analysis. After subsequent washing, the cells were labeled with anti-γH2AX pS139 antibodies (130-118-339) according to the manufacturer’s protocol. ET (1 μg/mL) was used as a positive control. In each sample, 10^4^ single cells were recorded and independent experiments were repeated three times. GraphPad Prism 9 was used for data visualization. Statistical significance between the solvent control and the tested cell populations was determined with the R program [42], using a linear mixed-effects model and its packages reshape [43] and nlme [44].

### 2.7. Changes in the Cell Cycle—Analysis by Flow Cytometry 

Changes in cell cycle distribution of cells in the tested cell populations were measured by flow cytometry. The HepG2 cells (400,000 cells/well) were seeded into 6-well plates (Corning Costar Corporation, Corning, NY, USA). ET (1 μg/mL) was used as a positive control. After the exposure, cells were harvested, fixed, stored, and washed as previously described for the γH2AX assay by Hercog et al. [40]. Washed cells were stained with the Hoechst 33342 dye (1:500 in 0.1% Triton X-100). Hoechst fluorescence was detected in the V1 (450/50 nm) channel (MACSQuant Analyzer 10), where 10^4^ single cells were recorded per experimental point in three independent experiments. The data were analyzed using the FlowJo V10 software package (Becton Dickinson, Franklin Lakes, NJ, USA) using the univariate Dean–Jett–Fox cell cycle model on single, debris-free cells. The evaluation of differences in the cell cycle distribution of cells in the test populations was performed by multinomial logistic regression and post estimation tests in Stata 15 (StataCorp LLC, College Station, TX, USA). An advanced classification technique, the multinomial logistic regression, enables the evaluation of the effect of different compound concentrations on the cell cycle distribution by predicting different outcome probabilities (G0/G1, S, and G2) from a set of independent variables. Changes in the cell cycle distribution were considered statistically significant when the following was true for any phase of the cell cycle: *p* < 0.001 and the average marginal effect (dy/dx) > 0.1 (multinomial logistic regression). 

## 3. Results and Discussion

**Figure 1:**
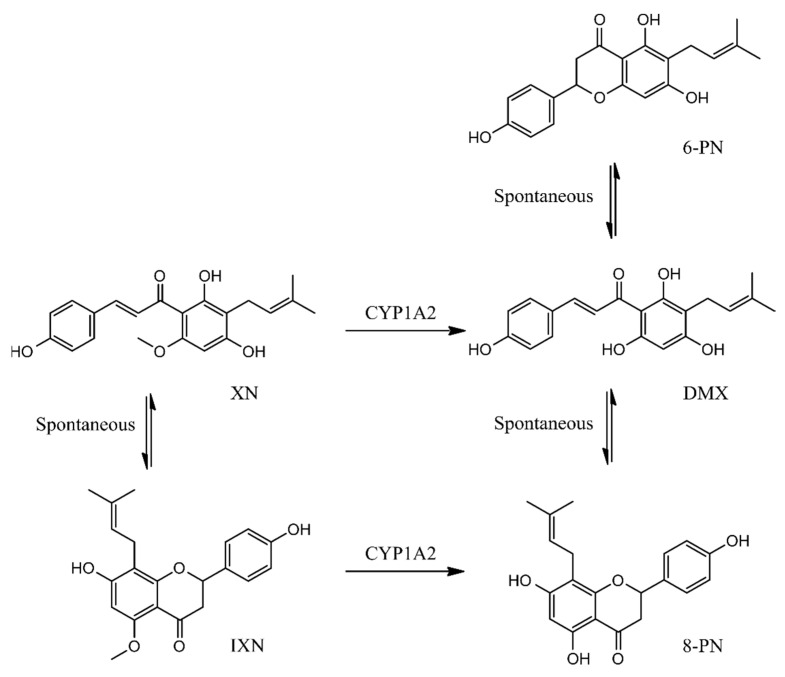
XN conversion into its derivatives: IXN, 6-PN, 8-PN, and DMX.

The prenylated flavonoid from hops XN has received significant attention in the last decades as it has been observed to exert a variety of beneficial health-related effects including anticarcinogenic activity *in vitro* and in *in vivo*, primarily through its antioxidative properties, the inhibition of CYP450 enzymes, and, hence, prevention of pro-carcinogen activation, and the promotion of detoxifying processes [7,12]. XN bioavailability is, however, limited and despite being the major prenylflavonoid in hops, it is present in relatively low amounts in beer, due to its isomerization during the brewing process [9]. In the present study, XN was evaluated for its chemoprotective properties against the carcinogenic mycotoxin AFB1, which, upon ingestion, is converted to the ultimate carcinogen AFBO and other hydroxylated metabolites by the CYP450 isoforms CYP3A4 and CYP1A2 [26]. XN is also metabolized by CYP450s (Figure 1). It is non-enzymatically converted to its flavanone isomer IXN, which can be further converted into 8-PN by CYP1A2 [9,45]. XN is also converted to desmethylxanthohumol (DMX) by CYP1A2, which can be further converted to either 8-PN or 6-PN (Figure 1) [9]. Not much is known about the chemoprotective properties of XN transformation products and their metabolites and since the conversion of XN may play a considerable role in its bioavailability and bioactivity, its derivatives IXN, 8-PN, and 6-PN were also included in the *in silico* analysis of their molecular interactions with AFBO.

### 3.1. Mechanistic Insights into Alkylation Reactions of XN, IXN, 8-PN, and 6-PN with the Carcinogenic Metabolite of AFB1-AFBO

**Figure 2:**
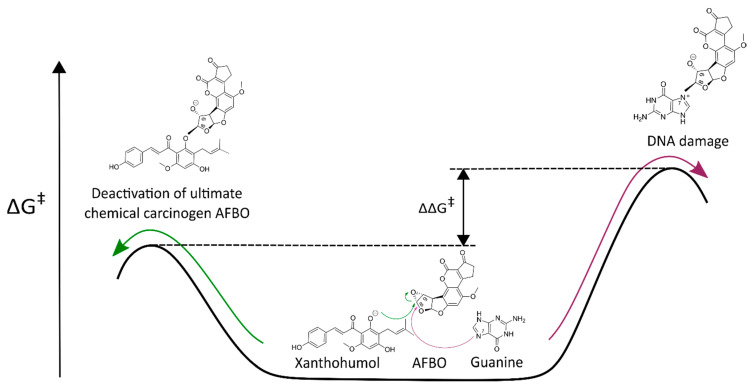
Competing reactions of the ultimate chemical carcinogen AFB1 exo-8,9-epoxide (AFBO) with xanthohumol (XN; green arrow) and guanine (violet arrow).

The kinetics of AFBO-XN, AFBO-IXN, AFBO-8-PN, and AFBO-6-PN binding, as well as AFBO-DNA adduct formation, were evaluated by the calculations of the ΔG^‡^ required for the reactions of XN, IXN, 8-PN, and 6-PN with AFBO, which were compared to the ΔG^‡^ required for the reaction of AFBO with guanine. A lower ΔG^‡^ represents a faster reaction and indicates preferential binding. We hypothesized that the ΔG^‡^ of the reaction between AFBO and XN (or its derivatives) is lower than the ΔG^‡^ of the reaction between AFBO and guanine (Figure 2).

**Figure 3:**
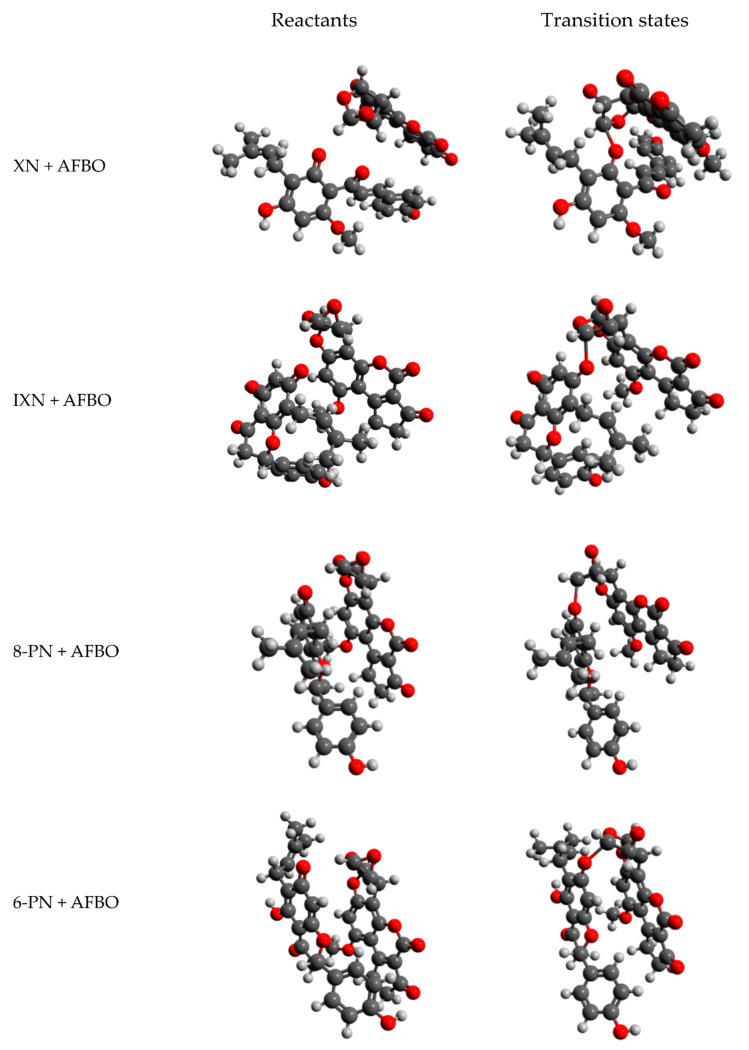
Visual representations of the reactant (**left**) and transition state structures (**right**) of the AFBO-XN, AFBO-IXN, AFBO-8-PN, and AFBO-6-PN complexes, obtained at the Hartree–Fock level of theory in conjunction with 6-311++G(d,p) basis set. The visual representations of reactant and transition states of the individual complexes clearly show the changes in orientation between the reactant and transition state structures as well as the change in molecular orientation after bonding.

From the visual representations of reactant and transition state structures collected in Figure 3, it can be observed that the epoxy ring of the ultimate chemical carcinogen AFBO opens, while the least-hindered carbon atom of the epoxy ring reacts with the most nucleophilic oxygen of XN, IXN, 8-PN, and 6-PN, resulting in the formation of a new covalent C-O bond, which corresponds to the *S_N_2* reaction mechanism.

The calculated ΔG^‡^ at the HF/6-311++G(d,p) level of theory for the alkylation reactions between AFBO and XN, IXN, 8-PN, and 6-PN, lowest vibrational frequencies of reactant states, imaginary frequencies of transition states, and corresponding distances between the reactive centers are summarized in the Supplementary Materials (Table S1). Moreover, the M06-2X/6-311++G(d,p) level of theory is also included to demonstrate that this modern hybrid functional with 54% HF exchange produced the same relative order of ΔG^‡^, albeit at lower values. The correctly optimized reactant state structure must have only real frequencies. The correct transition state structure must have exactly one imaginary frequency that corresponds to the reaction coordinate, representing the cleavage of the bond inside the epoxy ring of the ultimate chemical carcinogen AFBO and the formation of a new covalent bond between the nonchiral carbon atom of AFBO and the most nucleophilic oxygen atom of XN or its derivative. The obtained normal modes coincided with the formation of a chemical bond between the phenolic oxygen of XN, IXN, 8-PN, or 6-PN and the nonchiral carbon of the epoxy ring on AFBO. The proposed *S_N_2* substitution mechanisms for the formation of AFBO-XN (or its derivative) complexes are depicted in Figure A1 (Appendix A). 

As the experimental ΔG^‡^ for the evaluation of our computational results was determined in solution, a series of calculations with incorporated solvation effects was performed. Literature review revealed that the most accurate ΔG^‡^ for the alkylation reactions can be obtained with the HF/6-311++G(d,p) method in conjunction with SCRF and LD implicit solvation models [34,35,37,46,47]. Therefore, these methods were utilized to calculate the corresponding ΔG^‡^ for the reactions of AFBO with XN and its derivatives as well as the ΔG^‡^ for the reactions of AFBO with the most reactive DNA base guanine. In order to determine the best combination of the HF/6-311++G(d,p) method and solvation models (SCRF or LD), the calculated ΔG^‡^ for the reaction of AFBO with guanine was compared with the experimental ΔG^‡^ for the reaction between AFBO and guanine. 

Table 1 summarizes the results obtained with the SCRF and LD implicit solvation models at the HF/6-311++G(d,p) level of theory for the reactions of XN, IXN, 8-PN, 6-PN, and guanine with AFBO. For the evaluation of the computational results, the experimentally obtained ΔG^‡^ of the reaction between AFBO and guanine is also included [34].

The Hartree−Fock level of theory in conjunction with the flexible 6-311++G(d,p) basis set and the LD solvation model reproduced the experimental ΔG^‡^ of the reaction between AFBO and guanine in the most accurate way. The absolute difference between the experimental and calculated ΔG^‡^ with the LD solvation model for the reaction between AFBO and guanine was lower than 1 kcal/mol (0.85 kcal/mol), which represents an acceptable calculation error. ΔG^‡^ calculated with the LD solvation model is expected to be in very good agreement with the experimental ΔG^‡^ for the reactions of this chemical carcinogen with XN, IXN, 8-PN, and 6-PN. On the other hand, the HF/6-311++G(d,p) method in combination with the SCRF solvation model did not reproduce the experimental value for the reaction of AFBO with guanine within the acceptable calculation error. Based on the calculated ΔG^‡^, the same trend can also be expected for reactions of AFBO with XN, IXN, 8-PN, and 6-PN. The HF/6-311++G(d,p) method in combination with the LD solvation model was, therefore, chosen to evaluate the reactivity of XN and its derivatives with AFBO. 

From the results collected in Table 1, the following order of reactivity for XN and its derivatives with AFBO can be established: 8-PN < XN < IXN < 6-PN. The ΔG^‡^ obtained with the LD solvation model for the reaction of AFBO, with 6-PN is the lowest (13.14 kcal/mol). Therefore, the reaction of AFBO with 6-PN is the fastest. The ΔG^‡^ obtained with the LD solvation model is the highest for the reaction of 8-PN with AFBO (14.88 kcal/mol). The reaction of 8-PN with AFBO is, therefore, the slowest. The observed order of reactivity can be a consequence of the least sterically hindered structure in the case of 6-PN, which allowed the closest contact with AFBO, whereas the reactions of AFBO with XN, IXN, and 8-PN are more sterically hindered (Figure 3). 

Moreover, quantum-mechanical calculations at the HF/6-311++G(d,p) level of theory with the corresponding LD method predicted a significantly higher reactivity (>1 kcal/mol) of AFBO towards 6-PN than towards the most reactive DNA base guanine (by 1.11 kcal/mol), while the reactivity of IXN, XN, and 8-PN with AFBO is comparable to the reactivity of AFBO with guanine (Table 1). We can assume that the reaction between AFBO and 6-PN would occur faster than the reaction between guanine and AFBO, meaning that 6-PN could successfully prevent AFBO-induced DNA adduct formation. 6-PN, therefore, represents the primary scavenger of AFBO, although IXN, XN, and 8-PN also contribute to the overall scavenging activity. These findings indicate a novel possible mechanism underlying the chemoprotective effects of XN and its derivatives against AFBO, potentially contributing to the prevention of AFB1-specific DNA adduct formation.

DNA adduct formation is thought to be the main mechanism underlying AFB1 mediated carcinogenesis. After the reaction of AFBO with guanine, the formed AFB1-N7-guanine adduct, which is chemically unstable, breaks down into two types of secondary lesions. It can either form an open ring structure, producing an AFB1-formamidopyridine adduct (AFB1-FAPy) or the guanine residue can undergo depurination, resulting in an apurinic (AP) site [26]. Both the AFB1-N7-guanine and the AFB1-FAPy lesions are highly mutagenic, with mutation frequencies of 45 and 86%, respectively, predominantly inducing G to T transversion mutations [48]. DNA damage repair processes of AFBO-induced DNA adducts or AP sites, or DNA replication disruption or lesion bypass may result in DNA damage, DNA single-strand breaks (SSBs), and DSBs. These genotoxic endpoints were further examined *in vitro* along with the cytotoxicity assessment and cell cycle analysis for the evaluation of the chemoprotective effects of XN against AFB1.

### 3.2. Protective Effects of XN against AFB1-Induced Cytotoxicity and Genotoxicity In Vitro 

The chemoprotective effects of XN against the AFB1-induced cytotoxicity and genotoxicity were studied in the hepatocellular carcinoma cell line HepG2. This cell line is widely used in toxicological studies as one of the test systems of choice as it expresses the wild-type tumor suppressor TP53, has a known karyotype [49], and has retained the activity of several phase I and II metabolic enzymes involved in the metabolism and detoxification of xenobiotics [50]. This is particularly important as both AFB1 [26] and XN [45] are known to be metabolized by CYP450 enzymes.

**Figure 4:**
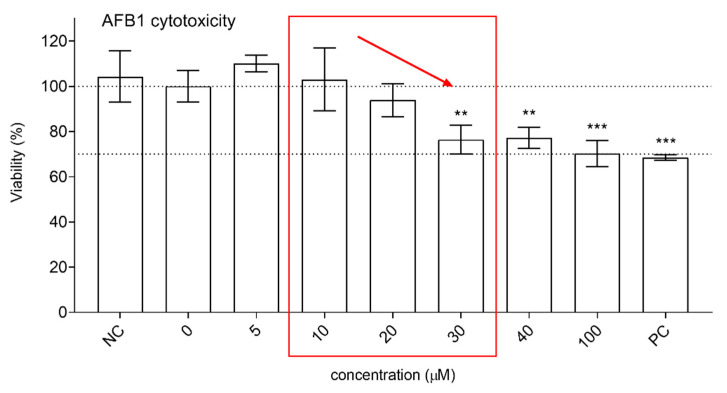
The effects of AFB1 (5–100 µM; 24 h exposure) on the viability of HepG2 cells (MTS assay) expressed in percentage of the solvent control (0; 1% DMSO—upper dashed line). NC is the negative control (growth medium). PC is the positive control—ET (25 μg/mL). The lower dashed line represents the 30% viability threshold. The asterisks (*) denote statistically significant differences (ANOVA and Dunnet’s multiple comparison test) between the solvent control and the AFB1 exposed cells (** *p* ≤ 0.01; *** *p* ≤ 0.001). The selected concentration range for further experiments is marked by the red square.

**Figure 5:**
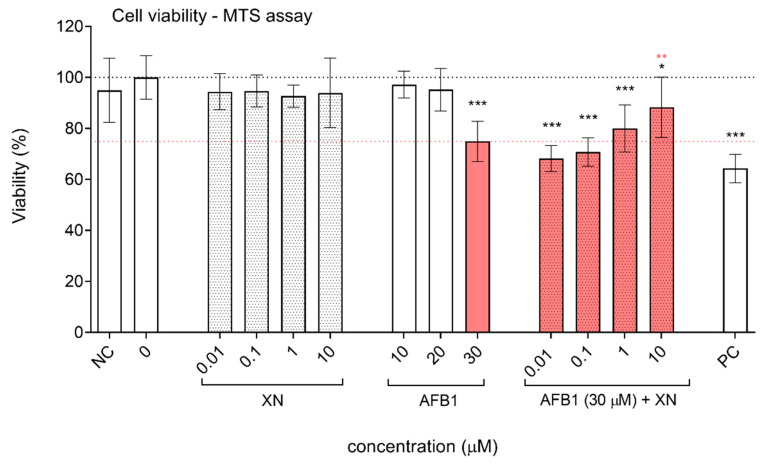
Protective effects of XN against AFB1-induced cytotoxicity. The effects of XN (0.01, 0.1, 1 and 10 µM), AFB1 (10, 20 and 30 µM), and the AFB1 (30 µM)—XN (0.01, 0.1, 1 and 10 µM) combinations on the viability of HepG2 cells after 24 h of exposure, presented as a percentage of the solvent control (0; 1% DMSO—upper dashed line). NC is the negative control (growth medium). PC is the positive control—ET (25 μg/mL). The lower red dashed line represents the average reduction in cell viability by AFB1 (30 µM). The asterisks (*) denote statistically significant difference (ANOVA and Dunnet’s multiple comparison test) between the solvent control and the AFB1, XN, and their combinations exposed cells (* *p* ≤ 0.05; *** *p* ≤ 0.001). The red dots (•) denote a statistically significant difference (ANOVA and Dunnet’s multiple comparison test) between the cells exposed to AFB1 (30 µM) and to the AFB1-XN combinations (•• *p* ≤ 0.01).

The concentrations of AFB1 and XN were chosen based on the cytotoxicity of the compounds towards HepG2 cells, which was evaluated using the tetrazolium-based MTS assay (Figure 4 and Figure 5) and previously published data [15]. Exposure of HepG2 cells to AFB1 at concentrations of up to 40 µM did not reduce cell viability by more than 30%, which is considered the limit value for genotoxicity assessments [51]. The concentrations 10–30 µM induced a dose-dependent decrease in cell viability (Figure 4); therefore, they were used in further experiments. 

AFB1 at 30 µM significantly reduced cell viability to 77.5%. The lower tested concentrations of AFB1 (5–20 µM) and XN alone (0.01–10 µM) had no statistically significant effect on HepG2 cell viability (Figure 4 and Figure 5). The addition of XN to AFB1 (30 µM) showed a slight dose-dependent protective effect against the cytotoxicity induced by AFB1 (Figure 5) that was statistically significant at 10 µM XN, indicating that XN protects HepG2 cells against AFB1-induced cytotoxic effects. 

**Figure 6:**
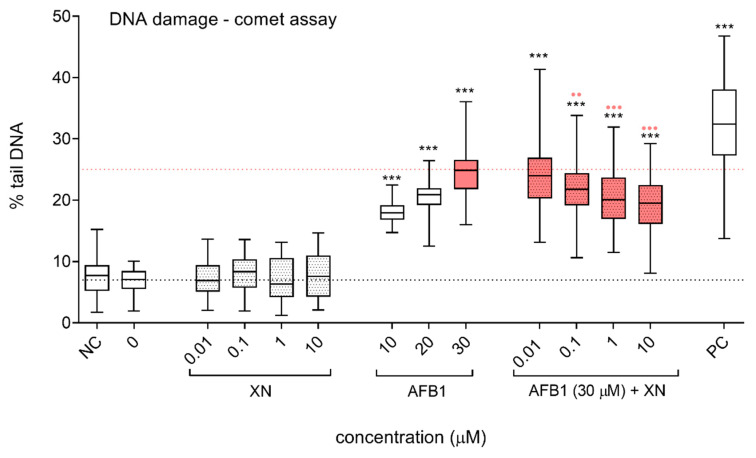
Protective effects of XN against AFB1-induced DNA strand breaks. The effects of XN (0.01, 0.1, 1, and 10 µM), AFB1 (10, 20, and 30 µM) and the AFB1 (30 µM)—XN (0.01, 0.1, 1, and 10 µM) combinations on DNA damage induction after 24 h of exposure. Data are expressed as % of “comet tail” DNA. 0 is the solvent control (1% DMSO).NC is the negative control (growth medium). PC is the positive control—BaP (30 μg/mL). The lower dashed line represents the median value of the % tail DNA in the solvent control. The upper red dashed line represents the median % of tail DNA in the cell population exposed to AFB1 (30 µM). The asterisks (*) denote a statistically significant difference (Kruskal–Wallis nonparametric test and Dunn’s multiple comparison test) between the solvent control and the AFB1, XN, and their combinations exposed cells (*** *p* ≤ 0.001). The red dots (•) denote a statistically significant difference (Kruskal–Wallis nonparametric test and Dunn’s multiple comparison test) between the cells exposed to AFB1 (30 µM) and to the AFB1-XN combinations (•• *p* ≤ 0.01; ••• *p* ≤ 0.001).

**Figure 7:**
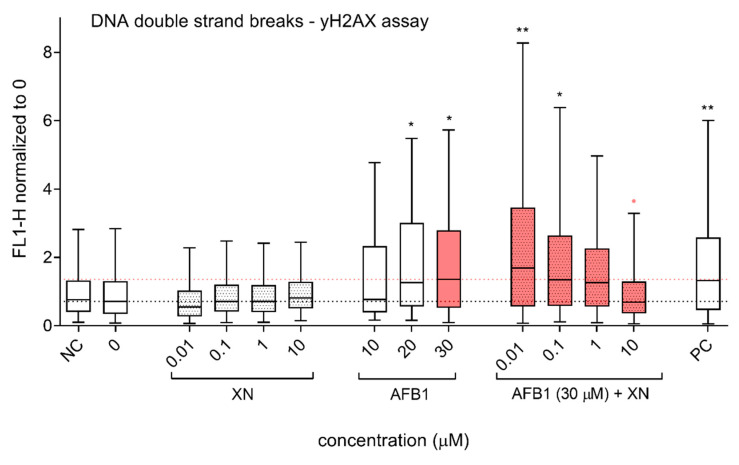
Protective effects of XN against AFB1-induced DNA double-strand breaks (DSBs). The effects of XN (0.01, 0.1, 1, and 10 µM), AFB1 (10, 20, and 30 µM) and the AFB1 (30 µM)—XN (0.01, 0.1, 1, and 10 µM) combinations on DNA DSB induction were assessed after 24 h of exposure. The data (fluorescence FL1-H) are normalized to the average fluorescent signal of the solvent control (0; 1% DMSO), and the lower dashed line denotes the median value of the relative fluorescence of 0. NC is the negative control (growth medium). PC is the positive control—ET (1 μg/mL). The upper red dashed line represents the median relative fluorescence in the cell population exposed to AFB1 (30 µM). The asterisks (*) denote statistically significant difference (linear mixed-effects model (lme), the R program) between the solvent control and the AFB1, XN, and their combinations exposed cells (* *p* ≤ 0.05, ** ≤ *p* 0.01). The red dots (•) denote a statistically significant difference (linear mixed-effects model (lme), the R program) between the cells exposed to AFB1 (30 µM) and to the AFB1-XN combinations (• *p* ≤ 0.05).

The protective effects of XN against DNA damage induction by AFB1 were assessed with the alkaline comet assay (Figure 6) and the detection of γH2AX induction using flow cytometry (Figure 7). The alkaline comet assay detects a diverse range of DNA lesions, including DNA SSBs and DSBs, alkali labile sites including apurinic/apyrimidinic (AP) sites, and SSBs resulting from incomplete excision repair [41]. AFB1 induced a statistically significant dose-dependent increase in those DNA lesions. XN did not induce DNA damage in HepG2 cells at the tested concentrations, after 24 h of exposure, while its addition to AFB1 significantly and dose-dependently reduced AFB1-induced DNA (Figure 6). In line with our results, XN (0.01–10 µM) has been previously reported to effectively prevent DNA strand breaks in HepG2 cells exposed to the mutagens formed in cooked meat, heterocyclic amines (HCAs), 2-amino-3-methylimidazo[4,5-f]quinolone (IQ) [15], 2-amino-1-methyl-6-phenylimidazo[4,5-b]pyridine (PhIP) [14], and 2-amino-3,8 dimethylimidazo[4,5-f]quinoxaline (MeIQx) [14], the polycyclic aromatic hydrocarbon (PAH) benzo(a)pyrene (BaP) [15], and the oxidative stress inducer *tert*-butyl hydroperoxide (TBHP) [15]. Furthermore, XN (0.01 µM) completely prevented IQ- and BaP-induced DNA damage, detected with the comet assay, in rat liver slices [16]. Similar observations were reported *in vivo* in rats, where XN reduced IQ-induced DNA strand breaks in colon mucosa cells and hepatocytes, at doses that could be reached after the consumption of high XN content beer [52]. Recently Pilcher et al. [17] conducted a placebo-controlled, human intervention trial, where DNA damage induction was analyzed in isolated lymphocytes exposed to liver homogenate (S9) activated carcinogens (BaP, IQ, and nitrosodimethylamine (NDMA)). The lymphocytes were isolated from participants before, during, and after two weeks of XN consumption (12 mg/day). The authors reported a substantial reduction of BaP- and IQ-induced DNA strand breaks, measured with the comet assay, after XN consumption and a moderate protective effect against NDMA. Additionally, they confirmed a reduction in BaP- and IQ-induced DNA DSBs, using the γH2AX assay, after 14 days of XN consumption.

DNA DSBs are considered the most deleterious form of DNA lesions, promoting genomic instability, and carcinogenesis if left unrepaired [53]. In the present study, DNA DSB induction was evaluated by measuring γH2AX formation using flow cytometry. γH2AX, the phosphorylated form of the histone H2AX, is a highly sensitive marker for DNA DSBs [54]. H2AX becomes phosphorylated on the serine residue 139 as a response to DNA DSB induction, forming foci adjacent to sites of DSBs [55]. This phosphorylation is abundant, fast, and adequately reflects the number of induced DSBs [56]. Our results showed that AFB1 (20 and 30 µM) significantly increased DNA DSB formation. The addition of XN dose-dependently reduced AFB1 (30 µM) induced DNA DSBs (Figure 7). XN at 10 µM completely reduced AFB1-induced DNA DSBs, reaching the control level.

**Figure 8:**
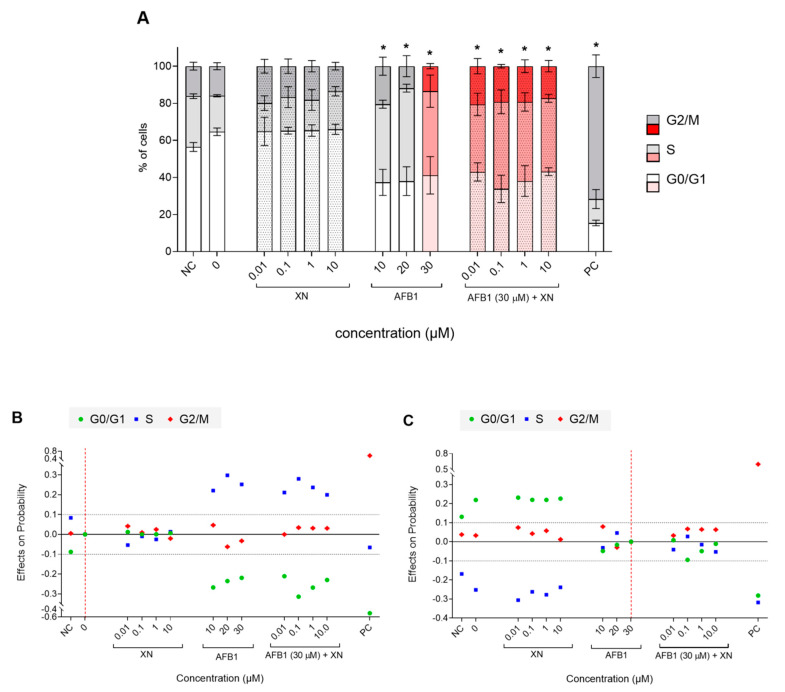
Protective effects of XN against AFB1-induced cell cycle arrest. The effects of XN (0.01, 0.1, 1, and 10 µM), AFB1 (10, 20, and 30 µM) and the AFB1 (30 µM)—XN (0.01, 0.1, 1, and 10 µM) combinations on the cell cycle after 24 h of exposure. (**A**) Distribution of cells (%), exposed to solvent (0) AFB1, XN, and AFB1−XN combinations, in the phases of the cell cycle (G0/G1, S, and G2/M). The asterisks (*) denote statistically significant difference (multinomial logistic regression)) between the solvent control (0) and the AFB1, XN, and their combinations exposed cells (*p* < 0.001 and the dy/dx > 0.1)). NC is the negative control (growth medium). PC is the positive control—ET (1 μg/mL). (**B**,**C**) show the effect of AFB1, XN, and AFB1–XN combinations on the cell cycle distribution compared to the solvent control (**B**) and 30 µM AFB1 (**C**), by predicting the probabilities of different outcomes (G0/G1, S, and G2/M) from a set of independent variables. The horizontal dashed lines denote a difference that is considered as significant, less than−0.1 and more than 0.1. The red, vertical dashed line denotes the base outcome, to which the other samples are compared for calculating the effects on probability.

DNA damage triggers the cellular response, including cell cycle arrest, during which DNA damage is being repaired, or cell death in case of severe unrepairable damage. Our results showed that AFB1 (10–30 μM) induced cell cycle arrest in the S phase of the cell cycle in HepG2 cells (Figure 8A). The average marginal effects, which demonstrate the effects of AFB1 (10–30 μM) on the predicted probability of each outcome (G0/G1, S, and G2/M), show that after the treatment with AFB1 (10–30 μM) the percentage of cells in the S phase was higher for 22, 30, and 25 percentage points, respectively, compared to the solvent control (0) (Figure 8B). This is consistent with previously published data reporting S-phase arrest in human bronchial epithelial cells [57], in the neuroblastoma IMR-32 cell line [58], and in HepG2 cells [59,60]. XN did not affect the cell cycle at the tested concentrations (Figure 8A,B); it also did not prevent or attenuate the AFB1-induced S phase arrest, which was confirmed by the average marginal effects on the predicted probability (Figure 8C). In the S phase, there are three checkpoints, two of which respond to errors in DNA replication (the replication checkpoint and S/M checkpoint). The third one (the intra-S-phase checkpoint) is induced in response to DSBs, preventing damaged cells to proceed with DNA replication [61]. Since the addition of XN at the tested concentrations could not completely prevent AFB1-induced DNA damage (Figure 6) it is possible that the remaining level of DNA damage still caused S phase arrest, with no difference that could be detected in the present study.

AFB1-mediated cytotoxicity, genotoxicity, and carcinogenicity primarily result from AFB1-specific DNA and protein adduct formation and reactive oxygen species generation. AFB1 and other liver and gastrointestinal pro-carcinogens such as PAHs and HCAs require CYP540-mediated bioactivation into ultimate chemical carcinogens. Hop flavonoids are known potent selective inhibitors of CYP450 enzymes [7]. XN inhibits the activity of CYP1A1 [16,62,63], CYP1B1, and CYP1A2 [63]. IXN, 8-PN, and 6-PN were also shown to inhibit CYP1A1 activity. IXN and 8-PN were reported to be even stronger inhibitors of CYP1A2 than XN, suppressing the activation of AFB1 [63,64]. Hence, the ability of XN and its derivatives to inhibit CYP450 enzyme activity, thereby, preventing AFB1 bioactivation, is probably the major mechanism undelaying their chemoprotective effects against AFB1-induced adverse effects.

XN has well-known antioxidative properties and it has been shown to inhibit the activation of pro-carcinogens and to promote detoxifying processes [7,11,12], which are all possible mechanisms behind the reduction of AFB1-induced cytotoxicity and genotoxicity observed in the present study. However, the results of our *in silico* experiments suggest an additional mechanism not considered before, namely, the direct binding of XN and its derivatives (IXN, 8-PN, and 6-PN) to the AFB1 metabolite AFBO, resulting in the reduction of AFBO-DNA adduct formation.

## 4. Conclusions

The present research presents novel data on the chemoprotective properties of XN and the possible underlying mechanism of action against the carcinogenic mycotoxin AFB1. The computational results highlight that XN and its derivatives IXN, 8-PN, and primarily 6-PN can function as AFBO scavengers and, therefore, potentially attenuate chemical carcinogenesis triggered by the ultimate chemical carcinogen AFBO. Our results also present strong evidence for the validity of the proposed *S_N_2* reaction mechanism and applicability of quantum-mechanical methods to reactions of polyphenolic scavengers with chemical carcinogens. *In vitro*, XN successfully reduced AFB1-induced cytotoxicity and genotoxicity in the metabolically competent hepatocellular carcinoma HepG2 cell line, further highlighting the chemoprotective activity of this prenylated flavonoid.

## Figures and Tables

**Table 1:** Activation free energies (ΔG^‡^) obtained at the HF/6-311++G(d,p) level of theory in conjunction with SCRF and LD implicit solvation models for the reactions of AFBO with XN, IXN, 8-PN, 6-PN, and guanine.

Polyphenolic Scavenger	Aflatoxin B1 exo-8,9-epoxide		
HF/6-311++G(d,p)	$\Delta G_{\mathrm{SCRF}}^{‡}{\;}^{a}$ [kcal/mol]	$\Delta G_{\mathrm{LD}}^{‡}{\;}^{b}$ [kcal/mol]	$\Delta G_{\exp}*$ [kcal/mol]
XN	21.06	14.70	
IXN	21.72	14.44	
8-PN	24.56	14.88	
6-PN	23.73	13.14	
Guanine	28.90	14.25	15.1 [34]

^a^ ΔG^‡^ obtained by the SCRF solvation model. ^b^ ΔG^‡^ obtained by the LD solvation model. * Experimental ΔG^‡^ for the chemical reaction between AFBO and guanine. The experimental value was used to determine the best combination of method, basis set, and solvation model for the theoretical calculations of the activation barriers for the reactions between AFBO and guanine, XN, IXN, 8-PN, and 6-PN, which are underlined.

## Data Availability

Data available upon reasonable request to the corresponding authors.

## Supplementary Materials

The following are available online at https://www.mdpi.com/article/10.3390/foods10061331/s1, Table S1: The obtained results for the reactions of AFBO with XN, IXN, 8-PN as well as 6-PN at the HF and M06-2X levels of theory in conjunction with 6-311++G(d,p) basis set.
