# Supplementary material for: Chemoprotective Effects of Xanthohumol against the Carcinogenic Mycotoxin Aflatoxin B1
Source: Foods. 2021 Jun 9;10(6):1331. doi: 10.3390/foods10061331 (PMC8230236; doi:10.3390/foods10061331)
Supplement: Supplementary file 1 [file foods-10-01331-s001.zip › foods-1235596-supplementary.pdf]

Supplementary Materials

Chemoprotective Effects of Xanthohumol against the Carcinogenic Mycotoxin Aflatoxin B1

Alja Štern[1,†], Veronika Furlan[2,†], Matjaž Novak[1], Martina Štampar[1], Zala Kolenc[2], Katarina Kores[2], Metka Filipič[1], Urban Bren[2,3*], Bojana Žegura[1*]

[1] Department of Genetic Toxicology and Cancer Biology, National Institute of Biology, Večna pot 111, Ljubljana, Slovenia
[2]Faculty of Chemistry and Chemical Technology, University of Maribor, Smetanova 17, SI-2000 Maribor, Slovenia
[3]Faculty of Mathematics, Natural Sciences and Information Technologies, University of Primorska, Glagoljaška 8, SI-6000 Koper, Slovenia
*Correspondence: bojana.zegura@nib.si (B.Ž.); Tel.: +386 5 923 28 62 (B.Ž.); urban.bren@um.si (U.B.); Tel.: +386-2-229 4421 (U.B.)

† Co-first author, these authors contributed equally to this work.

The calculated activation barriers at the Hartree-Fock (HF) and M06-2X levels of theory in conjunction with 6-311++G(d,p) basis set for the alkylation reactions of ultimate chemical carcinogen aflatoxin B1 exo-8,9-epoxide (AFBO) with xanthohumol (XN), isoxanthohumol (IXN), 8-prenylnaringerin (8-PN) as well as 6-prenylnaringerin (6-PN) *in vacuo* and solvated with Self-consistent reaction field (SCRF) or Langevin dipoles (LD) solvation models, imaginary frequencies of transition states, lowest vibrational frequencies of reactant states and the corresponding distances between the reactive centers are collected in Table S1.

Table S1: The obtained results for the reactions of AFBO with XN, IXN, 8-PN as well as 6-PN at the HF and M06-2X levels of theory in conjunction with 6-311++G(d,p) basis set.

Method/Basis set	$\Delta E^{\ddagger}$ [kcal/mol][a]	$\Delta\Delta G_{hydr}^{SCRF}$ [kcal/mol][b]	$\Delta G_{SCRF}^{\ddagger}$ [kcal/mol][c]	$\Delta\Delta G_{hydr}^{LD}$ [kcal/mol][d]	$\Delta G_{LD}^{\ddagger}$ [kcal/mol][e]	ω^{TS} [i cm^{-1}][f]	ω^{R} [cm^{-1}][g]	d^{TS} [Å][h]	d^{R} [Å][i]
				**Xanthohumol**					
HF/6-311++G(d,p)	21.06	-0.63	20.43	-6.36	14.70	205.55	5.09	2.04	3.12
M06-2X/6-311++G(d,p)	20.89	-0.77	20.12	-6.66	14.23	475.24	22.29	2.01	2.93
				**Isoxanthohumol**					
HF/6-311++G(d,p)	27.52	-5,8	21.72	-13.08	14.44	226.98	10.74	2.07	3.11
M06-2X/6-311++G(d,p)	27.26	-6.91	20.35	-13.11	14.15	473.29	23.49	2.02	2.92
				**8-prenylnaringerin**					
HF/6-311++G(d,p)	26.62	-2,06	24.56	-11.74	14.88	221.94	5.56	2.01	3.15
M06-2X/6-311++G(d,p)	22.89	-5.48	17.41	-8.51	14.38	482.86	9.89	2.00	3.13
				**6-prenylnaringerin**					
HF/6-311++G(d,p)	30.13	-6,4	23.73	-16.99	13.14	246.36	12.95	1.94	3.07
M06-2X/6-311++G(d,p)	23.19	-3.04	20.15	-10.53	12.66	481.91	19.58	1.91	2.95

[a]Gas-phase activation energy. [b]Relative hydration free energy: hydration free energy of the transition state minus hydration free energy of the reactant state obtained by the SCRF method. [c] Activation free energy obtained by the SCRF method. [d] Relative hydration free energy: hydration free energy of the transition state minus hydration free energy of the reactant state obtained by the LD method. [e] Activation free energy obtained by the LD method. [f] The imaginary frequency corresponding to the transition state. [g] The lowest frequency value corresponding to the reactant state. [h] The distance between the phenolic oxygen on xanthohumol or its derivative and the electrophilic nonchiral carbon in the epoxy ring of AFBO in the transition state structure. [i] The distance between the phenolic oxygen on xanthohumol or its derivative and the electrophilic nonchiral carbon in the epoxy ring of AFBO in the reactant state structure.
